# Big tobacco's dirty tricks: Seven key tactics of the tobacco industry

**DOI:** 10.18332/tpc/176336

**Published:** 2023-12-20

**Authors:** John Gannon, Katharina Bach, Maria Sofia Cattaruzza, Yael Bar-Zeev, Sarah Forberger, Biljana Kilibarda, Razieh Azari, Uzoamaka Okwor, Marta Lomazzi, Bettina Borisch

**Affiliations:** 1 World Federation of Public Health Associations

**Keywords:** tobacco, industry, FCTC, advertising, lobbying, corporate

## INTRODUCTION

The tobacco industry has been influencing public opinion and disrupting health policy through sophisticated and deceptive methods for decades. As evidence has mounted supporting the undisputed deadly effects of tobacco products, corporations have found ways to remain profitable. They have succeeded in attracting enough new smokers to support industry growth, despite the fact that over 8 million tobacco-related deaths occur annually. The sphere of influence of multi-billion-dollar tobacco companies extends to the fields of scientific research, politics, law, sport, education and the media. Members of the Tobacco Control Working Group of the World Federation of Public Health Associations (WFPHA) reviewed the literature and conducted research into key tactics used by the tobacco industry, producing written reports on seven of these: 1) Tobacco advertising, promotion and sponsorship, 2) Front groups, 3) Manipulating the media, 4) Funding scientific research, 5) Political lobbying, 6) Electronic alternatives as ‘harm reduction’, and 7) Legal challenges. Each tactic, including examples of their occurrence and recommendations on how to recognize and counteract them, are comprehensively explored in the casebook *Big Tobacco’s Dirty Tricks* which can be accessed at the link provided^1^. This article summarizes the seven tactics discussed in the casebook.

### Tobacco advertising, promotion and sponsorship (TAPS)

In 2019, the largest tobacco companies spent US$8.2 billion on advertising in the US alone^2^. Global progress has been made in restricting TAPS, supported by the World Health Organization (WHO) Framework Convention on Tobacco Control (FCTC)^3^. Effective implementations include regulating media advertising, point-of-sale display bans and plain packaging of tobacco products, among others^3^. As various forms of advertising are restricted, Big Tobacco adapts by finding loopholes and increasing investment in other domains^4,5^. This may be through marketing targeting specific populations such as young people with point-of-sale advertising and internet promotions^6,7^ and directly to female consumers with branding terms aimed at ‘stylish and feminine’ users, according to tobacco industry documents^8,9^. Tobacco sponsorship occurs in sports events such as Formula 1, and even in education where tobacco-sponsored schools are constructed, as in Project Hope in China^10,11^. Further, retailer incentive programs are implemented that allow cigarette manufacturers to pay for the control of product placement, pricing and promotion in stores^12^.

***Recommendations***

*Comprehensive advertising bans*

TAPS methods must be identified and counteracted by governing bodies, as any loopholes in legislation will be ruthlessly exploited by the industry^13^. Implementation of extensive bans on marketing tactics including retailer incentive programs are needed to reduce tobacco prevalence. Public health actors should establish counter-marketing strategies such as mass media anti-smoking campaigns. These have been shown to be effective, especially among young people^14,15^. FCTC Article 13 strongly denounces retailer incentive programs, but few countries have binding legislation to prevent it^16,17^.

*Plain packaging and health warnings*

Package design is an important component of branding. Many governments have introduced or are considering introducing plain packaging to limit this form of TAPS^18^. In 2012, Australia became the first country to legislate plain packaging for cigarettes, mandating that all packs have a standard size, color and include graphic health warnings^19^. FCTC Article 11 advises all countries to introduce health warnings on packages^20^.

### Front groups

Tobacco industry front groups are organizations or coalitions created and/or funded by tobacco companies. Group ties to the tobacco industry are usually hidden or minimized. Front groups commonly purport to represent a particular agenda, which is often aligned with the industry’s interests. Front groups work to create the illusion of public sympathy for certain issues (e.g. deregulation of the industry) and that a range of independent third parties support tobacco industry positions. The groups also aim to influence the opinions of leaders, legislators, regulators, health professionals and the general public^21^. Smokers’ rights groups, for example, are used to create grassroots networks that can be efficiently mobilized to oppose smoking restrictions^21^. Front groups can also be used by tobacco companies to lobby health organizations, while distancing the activity from the company itself.

***Recommendations***

*Remaining vigilant and reporting suspicions*

The activities of industry front groups greatly undermine tobacco control policy and practice. It is imperative to remain skeptical and carefully check sources and funding links when presented with data or opinions that are consistent with Big Tobacco objectives. Lists of known front groups can be found using the *Tobacco Tactics* ^22^ and *Expose Tobacco* ^23^ online resources, although these lists are by no means exhaustive. When reading research or opinions that appear suspicious, consider whether a front group is involved, check carefully for conflicts of interests and hidden ties to the industry, and report concerns to the relevant public health authorities.

### Manipulating the media

The tobacco industry has a long history of utilizing the media to promote cigarettes, and to sow doubt in the minds of the public about the harms of smoking^24^. In the 1950s, when evidence began supporting the link between smoking and lung cancer, newspapers were receiving significant revenue from tobacco advertisements and were reluctant to adequately inform readers of developments^25^. Since then, restrictions on tobacco advertising have increased significantly and the industry has become more nuanced in its approach in influencing what the media reports about their products. Creative methods used by the industry to get around restrictions include sponsoring the training and excursions of journalists, engaging in corporate social responsibility activities like public voluntary donations^26,27^, and paying influencers to promote products in the relatively unregulated social media domain^28^.

***Recommendations***

*Education and advocacy*

For World No Tobacco Day 2020, WHO launched a school toolkit focusing on protecting young people from exploitation by the industry^29^. They engaged social media sites, launching the TikTok challenge *#TobaccoExposed* and welcomed online partners like Pinterest, Tinder and YouTube to amplify messaging^30^. They called on all sectors to help to stop tobacco marketing tactics that prey on children and young people. Calls to action included social media marketing bans, schools refusing tobacco funding and prohibiting tobacco company representatives from speaking to students, celebrities and influencers rejecting sponsorship offers, and television services banning tobacco or e-cigarette use on screen. The campaign called on governments and financial sectors to take actions to divest of tobacco and the related industries.

*Social media regulation*

Compared to the structured media platforms that the tobacco industry has used over the past century, the digital marketing environment presents easier opportunities for companies to bend the rules and spread their message, particularly to younger more susceptible audiences. In 2019, Facebook and Instagram updated their tobacco policy, banning paid promotion of tobacco and vaping products, but this is difficult to enforce^31^.

*Mass media campaigns*

The media can be a positive force in tobacco control, and mass media campaigns as part of comprehensive tobacco control programs can inform the public, change behaviors and reduce smoking prevalence^32^. Research from the Centers for Disease Control and Prevention suggests that campaigns must reach >75% of their target audience for a duration of at least 18 to 24 months, to affect behaviour^33^. Emotional messages such as testimonials with compelling narratives, intense images and sounds, or graphic portrayals of negative health consequences, appear to be the most effective approaches^32,34^.

### Funding scientific research

Tobacco giants have invested historically on questioning the evidence of tobacco’s harms and trying to shift the blame for illnesses to other causes, although this has become more difficult to achieve over time^35^. Companies continue to fund scientists, academic journals and universities to produce research presenting favorable outcomes for products, including newer alternatives like e-cigarettes^36,37^. This way, consumers and policymakers can be misled about the health consequences of smoking or vaping^38^. A 2019 meta-analysis of 94 publications reported that 95.1% of studies with no financial conflicts-of-interest (COI) found potential harm caused by e-cigarettes, compared to just 7.7% of studies funded by the tobacco industry^39^. Millions of tobacco industry documents released following public settlement agreements, have revealed insights into companies manipulating research by commissioning publications with favorable results and conspiring to publicly question the effectiveness of policy interventions such as plain packaging^39-42^. Carrying out market research in the guise of scientific enquiry is another tactic that may be employed.

***Recommendations***

*Academic journal restrictions and transparency*

COI statements are mandated in academic literature but compliance can be an issue^43^. More substantial restrictions on industry involvement in research are necessary, such as penalties to authors who fail to disclose their relations to the tobacco industry^39^. All academic institutions should consider a strict policy of rejecting all articles with industry funding. Readers of literature should skeptically appraise any research with findings suggestive of positive outcomes from tobacco products.

*Enacting FCTC Article 5.3*

Article 5.3 recommends enacting public health policies for tobacco control, and preventing any influence from tobacco industry commercial interests^44^. The Article states: ‘In setting and implementing their public health policies with respect to tobacco control, Parties shall act to protect these policies from commercial and other vested interests of the tobacco industry.’^45^. Comprehensive and binding government policies could help to prevent the influence of scientific evidence funded by the tobacco industry^45,46^.

### Political lobbying

FCTC Article 5.3 forbids engagement between tobacco companies and public health policymakers, but political lobbying by the industry continues to occur. This may involve financial donations to political parties or candidates, or covering costs of travel, meals or hospitality, for example^47^. Threats may be used such as withholding economic support to apply pressure on politicians. Big Tobacco routinely supports conflicting roles; recruiting politicians to work for them or to lobby on their behalf, or securing positions in government for tobacco industry employees, thus giving them access to legislative processes^48,49^. The ‘revolving door’ effect describes instances in which politicians or civil servants move into roles with tobacco companies, or where current or former tobacco industry employees work within governments^50,51^.

***Recommendations***

*Advocacy and research*

Civil society organizations can investigate, report and expose examples of tobacco industry lobbying. Groups can undertake advocacy initiatives such as media campaigns and public communications. Tactics like political donations, economic threats and the ‘revolving door’ are less researched in low-income countries, and there is a need for more data in this regard.

*Compliance with FCTC Article 5.3*

Governments that have ratified FCTC must abide by their obligations and prevent tobacco industry interference in policymaking. Practical actions include mandating disclosure of all interactions with the tobacco industry, rejecting industry involvement in drafting tobacco control policies, banning financial contributions to political parties, and ensuring government institutions and individuals declare COI. The tobacco industry should be required to periodically disclose information on expenditure on marketing, lobbying, philanthropy, and political contributions. Finally, legislating for a ‘cooling off’ period, i.e. a specified amount of time to wait before a government official may take up a position in the private sector, or before employees in industries like tobacco can work in politics, would reduce the ‘revolving door’ effect^47^.

### Electronic alternatives as ‘harm reduction’

Battery-powered electronic nicotine delivery systems (ENDS) include e-cigarettes, which aerosolize a liquid solution when inhaled, and HTPs in which tobacco is heated but not burned^52,53^. With increasing TAPS restrictions and taxation, and declining cigarette sales in some regions, tobacco companies have identified these new technologies as booming markets in which to position themselves, particularly in high-income countries^52-54^.

ENDS offer several advantages for the industry, including less stringent regulation than conventional tobacco products^55^. Companies have been able to market them as healthier alternatives to smoking and an aid to cessation^56^. After decades of marketing lethal products to the public, Big Tobacco now present themselves as champions of ‘harm reduction’, striving to help smokers to quit cigarettes and transition to safer electronic alternatives^57,58^. The attention on ENDS also facilitates distraction of policymakers from classic tobacco control measures, moving the discourse away from cigarettes^59^.

ENDS sales facilitate the growth of tobacco companies by maintaining nicotine addiction and recruiting new users, particularly younger consumers. The global e-cigarette market was valued by Euromonitor International at over US$20 billion a year in 2019^55^. E-cigarettes and HTPs also help to rehabilitate the industry’s damaged image by giving the impression of a desire to improve smokers’ health^60^. The vocal involvement of the tobacco industry in the ‘harm reduction’ debate has polarized the public health community. Some believe that the companies must participate in regulation discussions to achieve anything, while others are convinced that, given its track record, the industry should not be allowed to influence policy in any form. Companies have been accused of using ENDS as a ‘trojan horse’ to infiltrate tobacco control decision-making processes^61^.

***Recommendations***

*Focus on the data*

Public health groups and policymakers must focus on the scientific data regarding safety and usefulness of ENDS as a smoking cessation tool, and not be affected by industry propaganda. Those who receive financial support from tobacco or vaping industries tend to emphasize the ‘harm reduction’ aspect of the products, but independent researchers have identified significant short-term health risks, and a knowledge gap about long-term effects^39,60^.

*Increased legislation*

Government policies to regulate ENDS as consumer products have not prevented their widespread availability. Legislation must be introduced comparable to what is in place for cigarettes, such as advertising restrictions, taxation and point-of-sale display bans. As of December 2022, 107 countries had bans or regulation of the distribution and sale of e-cigarettes^62^. Policies include minimum purchase age, consumption ban in public places, and regulations of nicotine concentrations, flavors and ingredients^63^. Traditional tobacco control measures must not be delayed or compromised due to the distracting effects of ENDS. Any reduction in these efforts is a success for the tobacco industry.

### Legal challenges

The tobacco industry has used litigation to contest all types of tobacco control measures, including tax policies, anti-tobacco advertisements, smoking bans, plain packaging, and even the formation of tobacco control authorities^64-68^. A common argument is that cigarettes are legal products, so punitive control measures breach international trade and intellectual property law^69^. It is not always necessary to win legal battles to declare the lawsuit a victory, as delaying and hindering progress in tobacco control translates into more profits for Big Tobacco^70^.

***Recommendations***

*Legal action against Big Tobacco*

There have been incidences of governments suing tobacco companies for harms to populations caused by their products, for example on the basis of misleading consumers, concealing information, and false marketing^71,72^.

*Alignment with WHO FCTC*

FCTC guidelines and other Conference of Parties decisions can support organizations facing legal challenges in a number of ways, including providing or strengthening the legal basis for a measure and supporting limitations on the exercise of commercial rights and interests. The FCTC can be used to demonstrate that a measure is evidence-based, supported by international practice or consensus, reasonable or proportionate, protects public health, and promotes human rights, and the rights to health and life. Alignment of government policies with FCTC is important in responding to lawsuits by Big Tobacco. When implementing tobacco control strategies, policymakers must ensure that their proposals are supported by FCTC, so they can rely on it as a robust defence if necessary^73^.

## CONCLUSION

The more informed we are about Big Tobacco’s tactics, the more effectively we can impose tobacco control measures. Recommendations put forward in relation to these seven tactics include comprehensive advertising bans, plain packaging and health warning legislation, remaining vigilant for possible industry front groups, education and advocacy, mass media campaigns, academic journal transparency, enacting FCTC Article 5.3, exposing political lobbying, preventing the ‘revolving door’ effect, improved regulation of ENDS, prioritizing data over propaganda in the ‘harm reduction’ debate, aligning policies with FCTC, and taking legal action against Big Tobacco where feasible. The recommendations are summarized in Table 1. It is imperative that health professionals and the general public have an understanding of the industry’s tactics to give us all the best opportunity to reduce consumption globally and prevent tobacco-related illness and death.

**Table 1:** Tactics and recommendations to counter them

*Tactics*	*Recommendations*
**Tobacco advertising, promotion and sponsorship**	Comprehensive advertising bans Point-of-sale display bans Prohibiting retailer incentive programs Counter-marketing strategies Plain packaging legislation Mandating health warnings on product packaging
**Front groups**	Remaining vigilant for possible front groups activity Checking lists of known front groups Reporting suspicions to health authorities
**Manipulating the media**	Education and advocacy campaigns Banning tobacco product usage on screen Social media regulation Mass media campaigns
**Funding scientific research**	Academic journal restrictions Compliance with conflicts-of-interest laws Scientific institution funding transparency Skepticism of publications with pro-tobacco research findings
**Political lobbying**	Prohibiting donations to political parties or politicians Advocacy campaigns Further research into lobbying practices Compliance with WHO FCTC Article 5.3 Preventing the ‘revolving door’ effect
**Electronic alternatives as ‘harm reduction’**	Focus on the data and independent research More legislation and regulation of e-cigarettes Ensuring ENDS do not distract from tobacco control measures
**Legal challenges**	Legal action against Big Tobacco Alignment of policies with WHO FCTC

## Data Availability

Data sharing is not applicable to this article as no new data were created.
